# CBD resistant *Salmonella* strains are susceptible to epsilon 34 phage tailspike protein

**DOI:** 10.3389/fmed.2023.1075698

**Published:** 2023-03-07

**Authors:** Iddrisu Ibrahim, Joseph Atia Ayariga, Junhuan Xu, Ayomide Adebanjo, Boakai K. Robertson, Michelle Samuel-Foo, Olufemi S. Ajayi

**Affiliations:** ^1^The Microbiology Program, College of Science, Technology, Engineering, and Mathematics (C-STEM), Alabama State University, Montgomery, AL, United States; ^2^The Industrial Hemp Program, College of Science, Technology, Engineering, and Mathematics (C-STEM), Alabama State University, Montgomery, AL, United States

**Keywords:** cannabidiol, resistance, *Salmonella*, antibacterial agent, phage

## Abstract

The rise of antimicrobial resistance is a global public health crisis that threatens the effective control and prevention of infections. Due to the emergence of pandrug-resistant bacteria, most antibiotics have lost their efficacy. Bacteriophages or their components are known to target bacterial cell walls, cell membranes, and lipopolysaccharides (LPS) and hydrolyze them. Bacteriophages being the natural predators of pathogenic bacteria, are inevitably categorized as “human friends”, thus fulfilling the adage that “the enemy of my enemy is my friend”. Leveraging on their lethal capabilities against pathogenic bacteria, researchers are searching for more ways to overcome the current antibiotic resistance challenge. In this study, we expressed and purified epsilon 34 phage tailspike protein (E34 TSP) from the E34 TSP gene, then assessed the ability of this bacteriophage protein in the killing of two CBD-resistant strains of *Salmonella* spp. We also assessed the ability of the tailspike protein to cause bacteria membrane disruption, and dehydrogenase depletion. We observed that the combined treatment of CBD-resistant strains of *Salmonella* with CBD and E34 TSP showed poor killing ability whereas the monotreatment with E34 TSP showed considerably higher killing efficiency. This study demonstrates that the inhibition of the bacteria by E34 TSP was due in part to membrane disruption, and dehydrogenase inactivation by the protein. The results of this work provides an interesting background to highlight the crucial role phage protein such as E34 TSP could play in pathogenic bacterial control.

## 1. Introduction

Cannabidiol (CBD), a constituent of *the Cannabis sativa* (hemp) plant is a non-psychoactive compound, which is a metabolite [2-(1R,6R)-6-isopropenyl-3-methylcyclohex-2-en-1-yl]-5-pentylbenzene-1,3-diol, (C_21_H_30_O_2_) with a molecular weight of 314.4636. It that plays essential roles in health and physiology. CBD has worldwide applications in medicine and possesses tremendous potential for pharmaceutical relevance, such as anti-microbial, antioxidants, anti-inflammatory, anti-cancer, and anti-convulsant potentials among many others. Several studies have reported the particularly enormous roles CBD plays in antimicrobial (anti-parasitic, antiviral, and antibacterial) infections (1–3). Fernandes et al. described in their studies how CBD was used to inhibit SARS-COV 2 infection in cells and in mouse models by blocking viral gene expression after gaining access to host cells. Their study relied on CBD and its metabolites 7-OH-CBD but did not include Tetrahydrocannabinol (THC). In other works, the anti-viral properties of CBD has been reported, particularly on SARS-COV2 (4–7). (8) examined the antibacterial properties of CBD extract against two strains of *Salmonella* (Typhimurium and Newington) and demonstrated that CBD was able to disrupt the membrane of the *Salmonella* spp. used in the study. Synergistic studies of polymyxin B and CBD co-therapy demonstrated strong antibacterial activities against *Acinetobacter baumannii* ATCC 19606 and other gram-negative bacteria (*Klebsiella pneumoniae* and *Pseudomonas aeruginosa*) (9). Some non-pathogenic bacteria such as gut microbiota is an integral aspect of human health, and while food and antibiotic consumption does affects the gut microbiota and sometimes provides an easy pathway for infections arising from foodborne pathogens (10), CBD has been demonstrated to boost enzyme activities of these gut bacteria (11, 12).

A whopping 115 million human infections and 370,000 deaths *per annum* are attributed to *Salmonella* infections globally. *Salmonella* is an important foodborne pathogen classified into 2,659 strains based on their surface antigens. *Salmonella* Typhimurium is known to be a pathogen of public health concern (13). *S*. Typhimurium is estimated to have resulted in 16–33 million infection cases and about 500,000–600,000 deaths globally *per annum* (14). Although, non-typhoidal *Salmonella* is a self-limiting disease, which is usually treated without the need for antibiotics, the need for antibiotics are required in immunocompromised individuals. Thus, though antibiotics play an enormous role in mitigating *Salmonella* infections, their efficacy is becoming futile due to the ability of bacteria to develop stringent resistance to antibiotics. This ontogeny has become a canker to health, and treatment of *Salmonella* infections. This foreclosure of *Salmonella* ontogeny means that novel antibiotic therapies must be fabricated to meet the urgent global health demand (15, 16). The economic and sanitary burden caused by antimicrobial resistance (AMR) is immense. An in-depth analysis conducted by a group of researchers revealed that the number of deaths caused by bacterial infections could be as high as 4.95 million annually (17–19). The researchers used a statistical model to analyze 471 million records from 16 different countries. They found that the mortality rate from bacterial infections could be as high as 1.27 million annually (20, 21). One of the most common causes of deaths associated with antimicrobial resistance is intra-abdominal infection. This contributes to the economic impact of the issue, as it costs the United States around USD 20 billion annually.

The natural enemies of bacteria are the bacteriophages. They can attack their target bacteria and are generally host-specific. This specificity attribute of phages allows for the maintenance of the human host’s microbiota if used as therapy (22). However, phages are self-limiting, easily cleared from the body, elicits strong immune response, thus making it problematic as a therapeutic agent. Additionally, these phages have narrow host range, thus compounding their use as antibacterial agents against a broad spectrum of bacterial infections. In phage therapy, it is crucial that the preparation of the stocks is free of bacterial toxins such as the bacteria LPS and even the host bacteria as a whole since these by themselves are agents of disease. The elimination of bacterial toxins and bacteria during the preparation steps thus presents a technical challenge and increases the production cost (23–26). To overcome these huddles, a much cheaper and safer approach could be the utilization of the phage’s hydrolases which the phage uses as its host attacking machinery.

The E34 phage belongs to the P22-like phages which have a uniquely short tailspike architecture, thus the name podovirus. Their tailspike protein is characterized by the presence of a globular head binding domain, a parallel beta-helix domain, and a beta-prism domain (27). Although the structures that are involved in viral adhesion and infection vary, a broad generalization of their properties can be made due to the presence of the β-helix domain. This unique domain is found in most of the viral and fungal structures that are commonly used in the development of human infectious diseases. Some of these include those of *Chlamydia, Helicobacteria, Borrelia*, and *Rickettsia* (28, 29). The parallel β-helix domains of other bacterial and fungal proteins are also known to be found in various surface and bacterial proteins (30–32).

Usually, parallel β-helix folds are not found in higher eukaryotes. However, these structures are known to exhibit high melting point (Tm), stability, and resistance to detergents at room temperature. Their role in the development of exterior virion structures that can endure harsh environmental conditions is also evidenced by their properties (30).

Most β-sheet proteins have been studied. The first known example of this is the pectate lyases from the *Erwinia* species, which are known to have a unique structure that allows them to infect plant cells (30, 31). These proteins are characterized by a long-arm elongated or solenoid-shaped structure. Most of the P22-like protein families, such as SF6, E15, and P22, have a unique structure that allows them to recognize and attach to host receptors. This structure also allows them to develop an LPS cleaving mechanism.

This study employs the tailspike proteins (TSP) of epsilon 34 (E34) phage in combination with CBD extract to understand its interactions with CBD-resistant *Salmonella* Typhimurium and *Salmonella* Newington. We hypothesized that phage protein-CBD extract combination exhibits antibacterial activity against *S*. Typhimurium and *S*. Newington.

## 2. Materials and methods

### 2.1. Media, chemicals, bacterial strains, and other reagents

All media, enzymes and oligonucleotides for PCR reaction, transformation and induction were purchased from New England Biolabs. Competent cells BL21/DE3 and Novablue cells were purchased from Novagen. *Salmonella* Typhimurium (the P22 phage host cells) *Salmonella* Newington (the E34 phage host cells) were received from other laboratories. The two strains of *Salmonella* were used in this study were lab coded as BV4012 and BV7004 representing *Salmonella* Typhimurium LT2 strain MS1868 (a kind gift from Dr. Anthony R. Poteete, University of Massachusetts) and *Salmonella* Newington as *S*. Newington (also known as *S. enterica* serovar Anatum var. 15+ strain UC1698, a kind gift from Dr. Sherwood R. Casjens, University of Utah) respectively. pET30a-LIC vector was purchased from Novagen, urea, sodium chloride, ammonium sulfate, and all other chemicals used in this research were of HPLC grade.

### 2.2. E34 TSP expression and purification

The transformants were grown on LB agar medium, which was premixed with 50 mg/ml of kanamycin antibiotic which serve to select for only transformers, non-transformers are killed by the 50 mg/ml kanamycin. A final concentration of 1 mM isopropyl-β-D-thiogalactopyranoside (Sigma) was used for induction of E34 TSP expression. The transformants were grown on LB agar medium, which was premixed with 50 mg/ml of kanamycin antibiotic which serve to select for only transformers, non-transformers are killed by the 50 mg/ml kanamycin. A final concentration of 1 mM isopropyl-β-D-thiogalactopyranoside (Sigma) was used for induction of E34 TSP expression. The initial cloning of the E34 TSP gene into Pet30a-LIC has been described elsewhere (33), however, the initial clone contained an extra 43 amino acids. In this work, the extra 43 amino acids have been removed so as to maintain the wild type E34 TSP only. Verification of a cloned insert was obtained by PCR analysis *via* 0.8% agarose gel electrophoresis using gene-specific primers (Forward primer; 5-pET-E34–5’-GACGACGACAAGATGACAGACATTACAGCC-3′. Reverse primer; 3-pET-E34–5’-GAGGAGAAGCCCGGTTCAAGACCAATACTC-3′).

BL21/DE3 cells containing the pET30a-LIC with the E34 TSP insert were streaked on Luria agar plates containing kanamycin. Streaked plates were incubated at 37°C in Precision Economy incubator overnight. This vector contains a 6HIS region that can be targeted for HIS-tag purification. Single colonies were selected and grown in LB broth primed with Kanamycin in a MaxQ 4,450 incubator (Thermo Scientific) fitted with a shaker running at 121 rpm. At mid-log, IPTG (Sigma) was added to a final concentration of 1 mM IPTG to induce cells. An uninduced control sample served as control for subsequent protein analysis. After 6 h, the bacteria were pelleted at 10,000 rpm in an Avanti J XP centrifuge fitted with JA14 rotor chilled at 4°C. Pelleted cells were then re-suspended in lysis buffer consisting of 50 mM Tris at pH7.4, 5 mM MgCl_2_, 0.1 mg/ml lysozyme, 0.1 mg/ml DNase, 0.05 mg/ml RNASE, 0.2 mg/ml DTT and subjected to three cycles of freeze–thaw–freeze. Samples were then centrifuged at 17,000 rpm for 30 min and the supernatant decanted into 50 ml tubes and stored at−20°C as the E34 TSP lysate. The fractionation of E34 TSP was then carried out using FPLC (GE/Amersham Biosciences- AKTA) connected to a desktop computer Pentium 4 running UNICORN software. In brief, 5 ml Cobalt-NTA FPLC columns (Co-NTA) was used in the FPLC fractionation of the E34 phage TSP at pressure of 0.27 MPa, flow rate of 1 ml/min. The samples that fell under the curve (7–8) were pooled together and concentrated using Amicon Ultra concentrators (Millipore). The gradient of 4.0 ml (100%) of buffer B served as the mobile phase. The buffer B consisted of 20 mM phosphate, 400 mM NaCl, 250 mM imidazole solution, pH 7.4. Then 3 fractions consisting of 1.0 ml each were collected and pooled together and fractions enriched to the desired concentrations using Amicon concentrators (MilliporeSigma). Purified samples were then run on 10% SDS PAGE to determine the purity of the protein.

### 2.3. Cannabidiol extract stock preparation and serial dilutions

The CBD extract hemp variety ‘Suver Haze’ CBD extract stock was obtained from Sustainable CBD LLC., and the extraction process has been published elsewhere (8). Valizadehderakhshan et al. have demonstrated other excellent and current method for obtaining CBD such as refining Cannabidiol using Wiped-Film Molecular Distillation (34). The stock was diluted with EtOH and vortexed to a final concentration of 50 mg/ml CBD extract and 4% EtOH. This was further diluted serially with 4% EtOH to produce our working concentrations of 250 μg/ml, 125 μg/ml, 62.5 μg/ml, 31.25 μg/ml, 15.62, and 7.81 μg/ml of CBD extract.

### 2.4. Creating CBD resistant strains

To investigate the antimicrobial activity mechanism of CBD, we generated CBD resistance following a procedure published by (35). *S*. Typhimurium strain BV4012 and *S*. Newington strain BV7004 were subjected to CBD resistance development by growing them for an extended time (7 days) in media supplemented with CBD extract. Initially, low doses of CBD extract (1–5 μg/ml), followed by selecting resistant colonies and growing them in CBD extract concentrations of 10–50 μg/ml. Finally, resistant colonies from these were plated on LB agar supplement with 50 μg/ml of CBD extract. Colonies formed were then grown again in LB broth to reach log phase. Resistant strains were then pelleted and resuspended in 1X PBS containing 50% (wt/vol) polyethylene glycol 8,000 (PEG 8000) (Promega Corporation, United States), 50 mM Mg2+ at pH 7.4. Finally, resistant bacterial samples were aliquoted into microcentrifuge tubes and stored at−80°C until use. Subsequently, CBD resistant strains of *S*. Typhimurium and *S*. Newington were restreaked on LB agar.

### 2.5. Assessments of CBD extract, E34 TSP, and their combination treatment of *Salmonella* spp.

Serial dilutions of CBD extract stock solutions were carried out to obtain the final concentrations of 250, 125, 62.5, 31.25, 15.62, and 7.81 μg/ml. Two *Salmonella* strains BV4012 (*S*. Typhimurium) and BV7004 (*S*. Newington) were grown in LB broth to logarithmic growth phase and diluted to OD_600_s of approximately 0.08 and 0.25. The CFUs were determined *via* plating on LB agar to consist of concentration of approximately of 1 × 10^4^ and 1 × 10^8^ CFU/ml. Then, aliquots of 100 μl of the bacterial cells were seeded in a 96-well microtiter plate (Fisherbrand™, Fisher Scientific, Fair Lawn, NJ, United States) at a density of 1 × 10^4^ per mL to mimic early log growth phase of the bacteria, or at a density of 1 × 10^8^ per mL to mimic log phase. Then, 50 μl of each solution of the protein, or the CBD extract or the combinations was added to the bacterial cells. The experimental groups were treated with 250, 125, 62.5, 31.25, 15.62, and 7.81 μg/ml concentrations of CBD extract, or 44.5, 22.25, 11.12, 5.56, 2.78, 1.39 μg/ml of E34 TSP, or the combinations of CBD extract and E34 TSP in varying concentrations. The controls consisted of dH_2_O group (negative control) and 2% SDS group (positive control) (see Table 1). The samples were incubated at 37°C for set time points. The growth kinetics of bacteria were determined by reading their optical densities at wavelength of 600 nm using SpectraMax plate reader [(Molecular Devices SpectraMax® ABS Plus) (Molecular Devices LLC., San Jose, CA, United States)]. The experiments were carried out in triplicates.

**Table 1:** Treatments.

Treatments	Concentrations/μg/mL	Remark
E34 TSP	44.5	44.5 to 11.25 μg/ml used in monotreatment analysis for early and late log phases of the bacteria.
	22.25	
	11.12	
	5.56	44.5 t0 1.39 μg/ml used in dose dependent treatment of the bacteria.
	2.78	
	1.39	
CBD	250	250 to 7.81 μg/ml CBD was used in the dose dependent treatment analysis CBD against the bacteria
	125	
	62.5	
	31.25	
	15.62	
	7.81	
E34 TSP + CBD	44.5 E34 TSP + 250 CBD	44.5 to 11.25 μg/ml used in combination treatment with 250 μg/ml of CBD.
	22.25 E34 TSP + 250 CBD	
	11.12 E34 TSP + 250 CBD	
Controls	LB/dH_2_O/SDS	

### 2.6. Membrane integrity assessment *via* propidium iodide and SYTO-9 staining

To assess the capacity of CBD extract, E34 TSP and CBD extract-E34 TSP combination to disrupt the cytoplasmic membrane integrity, the membrane impermeable fluorescent DNA intercalating dye Propidium iodide (PI) and SYTO-9 were employed which is a quick and accurate method to determine the proportion of dead and live cells in cell cultures. The fluorescence produced by the propidium iodide (PI) when it binds to DNA is used to identify dead cells (36). However, live cells with intact membranes present a barrier to PI, thus only cells with disrupted membranes can be stained with PI. Hence, this is used in assessing membrane integrity and cell death since dead cells will allow the penetration of PI into their cytoplasm. Fluorescence microscopy was utilized to observe the effects of CBD extract, or E34 TSP, or the combination of CBD extract and E34 TSP treatment on *Salmonella* cells. CBD resistant strains of *Salmonella*–strains *S*. Typhimurium and *S*. Newington–were grown to mid-log phase, diluted to cell concentration of 1 × 10^8^ and then treated with CBD extract, E34 TSP, or the combination of CBD extract and E34 TSP. Two controls, dH_2_O and 2% SDS served as the negative and positive controls, respectively. Samples were then stained with 1 X SYTO-9 and 40 μg/ml of propidium iodine and left at room temperature for 25 min, covered with aluminum foil. Samples were then observed using an EVOS FLC microscope (Life Technologies Corporation, Carlsbad, CA, United States).

### 2.7. Assessment of membrane lysis *via* genomic DNA migration

Bacteria cell membrane plays several crucial functions; however, the most fundamental function is to act as a storage sac for all the cytoplasmic content. Thus, if lysed, the cytoplasmic content which consists of the bacterial proteins and nucleic acid will leak. Taking advantage of this important role of cytoplasmic membrane, we hypothesized that the genomic materials of lysed cells will migrate in agarose gel matrix, whereas bacteria with intact cell membranes will retain their genomic material and thus block the nucleic acid from migrating through the agarose gel’s matrix when subjected to electrophoresis. To investigate the ability of E34 TSP, CBD extract or CBD extract-E34 TSP treatment to cause lysis of bacteria membrane, we pelleted 500 μl of *S*. Typhimurium that was previously growing at mid-log phase and resuspended the bacteria in 500 μl of 1X PBS. 100 μl of the bacteria sample was aliquoted into centrifuge tubes and 100 μl of 44.5 μg/ml E34 TSP, or 22.25 μg/ml of E34 TSP, or 250 μg/ml of CBD extract, or 125 μg/ml of CBD extract were added to the bacteria samples and incubated for 1 h. Sample of the resuspended bacteria which did not receive the CBD or E34 TSP treatments but rather media only served as control. Subsequently, 30 μl of each treatment sample was loaded into a 0.8% agarose gel and run for 75 min at 90 volts. Gel was then stained with Ethidium bromide and DNA bands visualized using ChemiDoc XRS imager.

### 2.8. Bacterial dehydrogenase activity assay

The combined dehydrogenase enzymatic activity of *Salmonella* spp. was assayed to assess the effect of CBD extract, E34 TSP or CBD extract-E34 TSP combination treatment on the bacterial cells in culture. In brief, *Salmonella* cells growing at mid-log phase were diluted to an OD_600_ of 0.2. Then, 100 μl of the bacterial sample was placed into each well of a 96-microplate and treated to varying concentrations of CBD extract, E34 TSP or CBD extract-E34 TSP. Then 50 μl of the non-toxic resazurin was added to each well. The conversion of resazurin to resorufin produced a sharp color change. The color change was monitored using SpectraMax plate reader (Molecular Devices SpectraMax® ABS Plus) (Molecular Devices LLC., San Jose, CA, United States) and absorbance readings recorded at 590 nm (37).

### 2.9. Statistical analysis

Two different *Salmonella* species were used for these experiments, and the experiments were carried out in triplicates, and results are presented as means ± SEM, *p*-values lower or equal to 0.05 were considered significant using student paired *t*-test. All statistical analyzes were performed, and graphs were plotted on Microsoft Excel (Microsoft 2010), microscopic images were processed using ImageJ (an opensource NIH software).

## 3. Results

### 3.1. Verification of E34 TSP gene insert in PET30 a-LIC vector and SDS PAGE analysis of expressed E34 TSP

**Figure 1:**
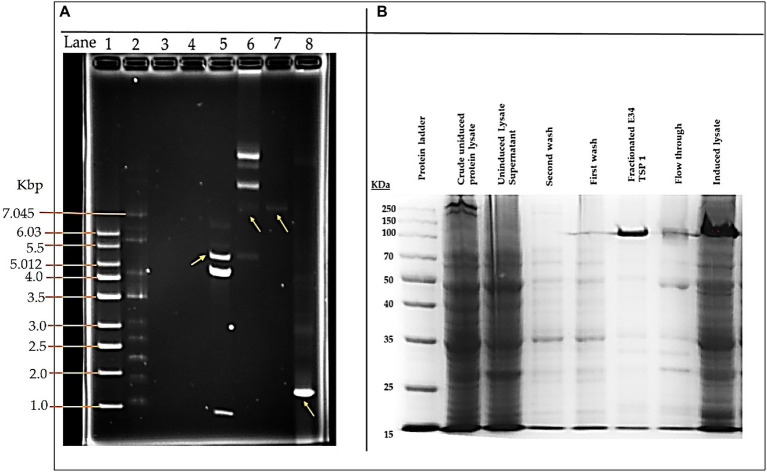
pET30a-LIC-E34 TSP clone verification and E34 TSP protein purification analysis. **(A)** Analysis of pET30a-E34 TSP clone in a 0.8% agarose gel. **(B)** Analysis of E34 TSP lysate and fraction using 10% SDS PAGE. Lane 1 = 1 kb DNA ladder, from Millipore Sigma. Lane 2 = NEB supercoil ladder; New England BioLabs. Lane 3 = miniprep for pET30a-E34 DNA in non-transformed cells. Lane 4 = miniprep for pET30a-E34 DNA in unsuccessfully transformed cells. Lane 5 = miniprep for pET30a-E34 DNA in successfully transformed cells; Nde1 digested pET30a-E34 DNA. Lane 6 = miniprep for pET30a-E34 DNA in successfully transformed cells; undigested pET30a-E34 DNA. Lane 7 = E34 WT DNA. Lane 8 = PCR product from pET30a-E34. **(B)** SDS-PAGE analysis of E34 TSP protein induced by IPTG, samples separated by 10% polyacrylamide gel, and visualized *via* Coomassie brilliant blue R-250. Induction of the E34 TSP was confirmed *via* the SDS PAGE. Comparing the induced lane and uninduced lane, it is observed that there was an overexpression of E34 TSP as indicated by the thicker band in the induced lane than the uninduced lanes.

**Figure 2:**
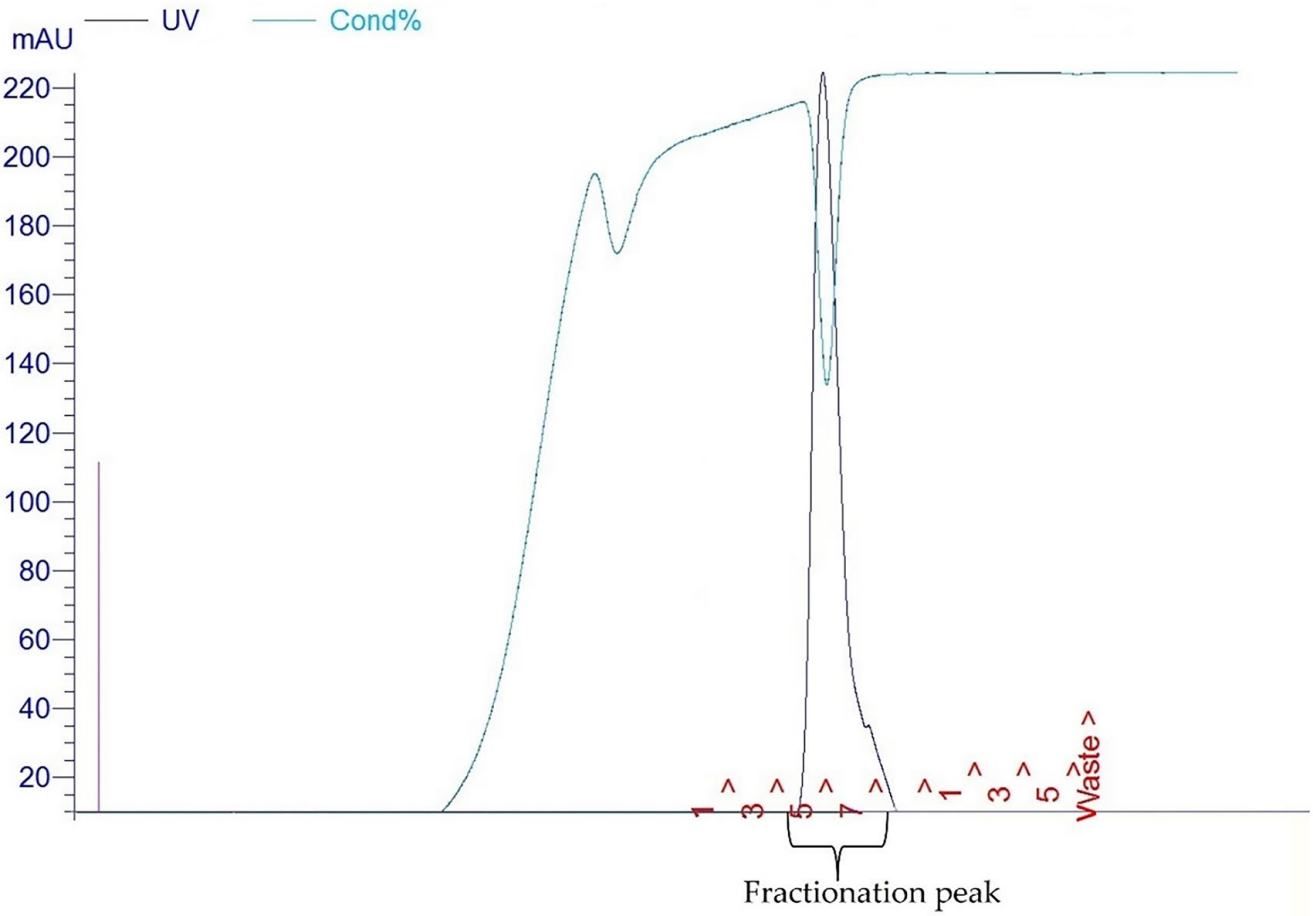
Fast protein liquid chromatography chromatograms (FPLC) of E34 TSP.

As shown in Figure 1A, lane 8, our PCR product produced the 1.818 kbp size insert which is the size of E34 TSP gene. In Figure 1B, the expressed E34 TSP migrated to a size consistent with its trimeric molecular weight size (see lane induced lysate and fractionated E34 TSP 1) this unusual trimeric migration property of E34 TSP has been well documented, and has been implicated on the non-covalent interactions between the three subunits of the protein (38–40), similar observation is noted of most other P22-like phages’ tailspike proteins (41–43). The purification process carried out *via* FPLC as shown in Figure 2 ensured the removal of over 90% contaminating proteins as shown in the fractionated E34 TSP1. Induction of E34 TSP was achieved as a thicker band could be visibly seen in the induced lane as compared to the uninduced lane where the E34 TSP band could barely be observed.

### 3.2. Effect of E34 TSP and CBD extract treatments on *Salmonella* Typhimurium and *Salmonella* Newington growth at early and late log phases

**Figure 3:**
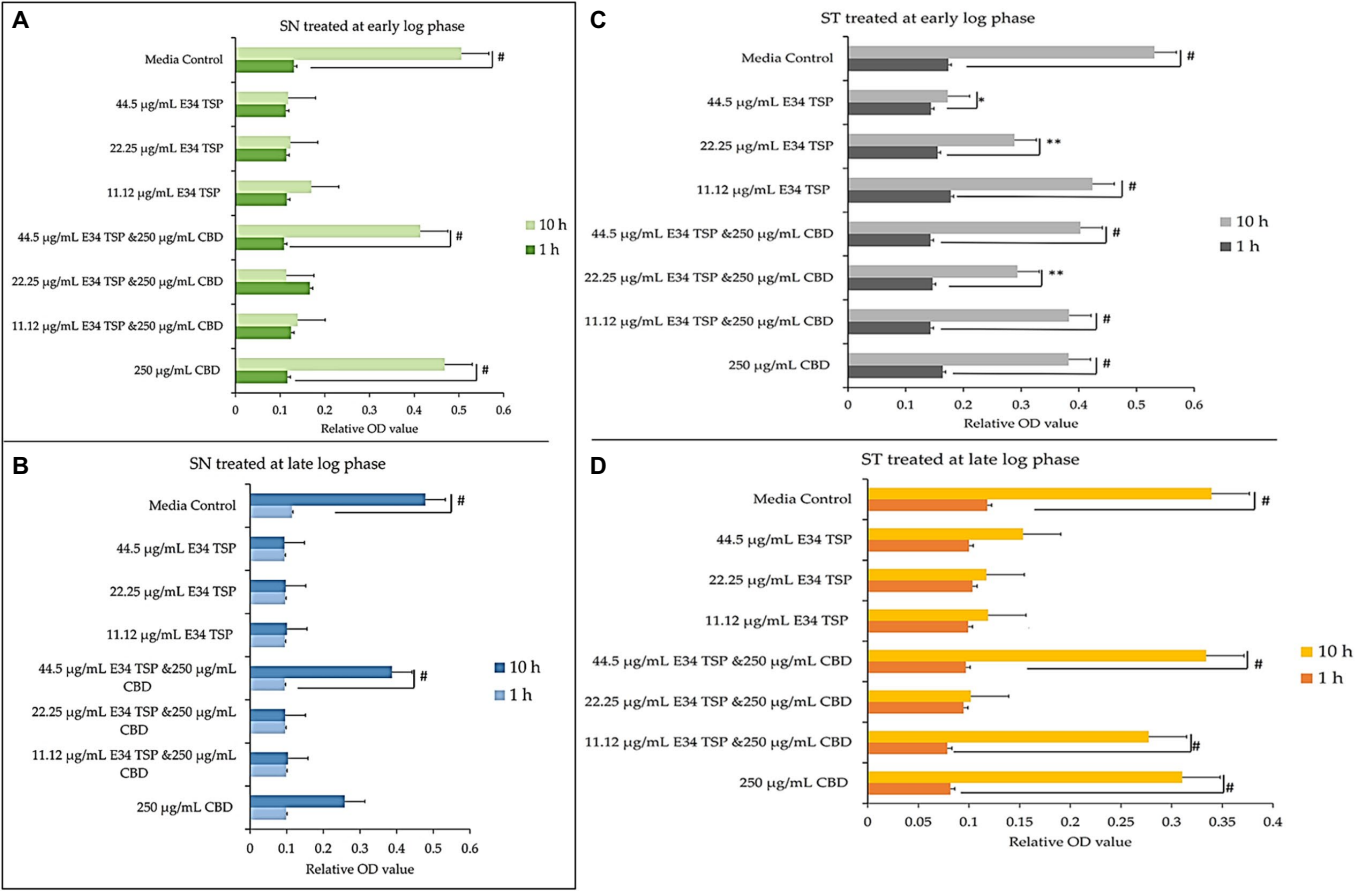
**(A,B)** Effect of CBD extract /E34 TSP treatment of *S*. Newington in both early and late log phase of the bacteria species. Studies were initiated an initial OD_600_ of approximately 0.1. **(A)** CBD extract and E34 TSP treatment to *S*. Newington (SN) at early log phase. # value of *p* ≤ 0.00354. **(B)** CBD extract and E34 TSP treatment to *S*. Newington (SN) at late log phase. Data shown are from three independent experiments expressed as means ± SEM. # value of *p* ≤ 0.039. **(C,D)** Effect of CBD extract and E34 TSP treatment of *S*. Typhimurium in both early and late log phase of the bacteria species. **(C)** CBD extract and E34 TSP treatment to *S*. Typhimurium (ST) at early log phase. # value of *p* ≤ 0.0001, * and ** value of *p* ≤ 0.025. **(D)** CBD extract and E34 TSP treatment to *S*. Typhimurium (ST) at late log phase. Studies were initiated an initial OD_600_ of approximately 0.1. Data shown are from three independent experiments expressed as means ± SEM. # value of *p* ≤0.0103.

At log phase, most bacterial disease symptoms begin to surface, and it is the growth phase which shows most dramatic changes in both bacteria number and disease severity. To assess the effect of E34 TSP, CBD extract and CBD extract-E34 TSP combination treatments on *S*. Typhimurium and *S*. Newington growth at early and late log phases, cells growing at early or late log OD_600_’s were subjected to E34 TSP, CBD extract or CBD extract-E34 TSP combination treatments as depicted in Figure 3.

As shown in Figures 3A,B, treatment of *S*. Newington with CBD extract, E34 TSP and the combination of the two produced interesting findings. We observed that the relative OD_600_ of E34 TSP treated samples were generally lower than the combined treatment for the early log-phase at the 10 h time point of culture. The data revealed a decrease in bacterial relative OD_600_s in both E34 TSP treated samples than the combination treatment. The exception however was observed in the 22.25 μg/ml E34 TSP and the 250 μg/ml CBD extract combination treatment which depicted a drastic increase in relative OD_600_. The 250 μg/ml CBD extract treatment only produced relative OD_600_ comparable to the media control. In the 44.50 μg/ml E34 TSP and the 250 μg/ml CBD extract combination treatment, we observed approximately triple jump in relative OD_600_ as compared to the 44.5 μg/ml E34 TSP monotreatment of *S*. Newington Figure 3. Similar trends were observed in the late log phase treatment of *S*. Newington to E34 TSP and CBD extract as depicted in Figure 3B.

In Figures 3C,D, treating *S*. Typhimurium (ST) with E34 TSP showed a generally lower relative inhibition characteristic after 10 h (especially at the higher concentrations) than the E34 TSP and CBD extract combination treatments. For instance, it was observed that at higher concentrations of E34 TSP (44.5 μg/ml) alone, there was a drastic reduction in relative OD_600_, however its combination with CBD extract (i.e., 44.5 μg/ml E34 TSP and 250 μg/ml CBD extract) performed poorly against the bacteria at both early and late log phases. Thus, when E34 TSP was combined with CBD extract at 44.5 μg/ml and 250 μg/ml respectively, the treatment performed poorly against the bacteria. Treatment of the bacteria with CBD extract alone did not reduce the relative OD_600_ in the early and late growth phases of the bacteria.

### 3.3. Immunofluorescent analysis of E34 TSP, CBD extract, and CBD extract-E34 TSP combination treatment on *Salmonella* Typhimurium and *Salmonella* Newington

**Figure 4:**
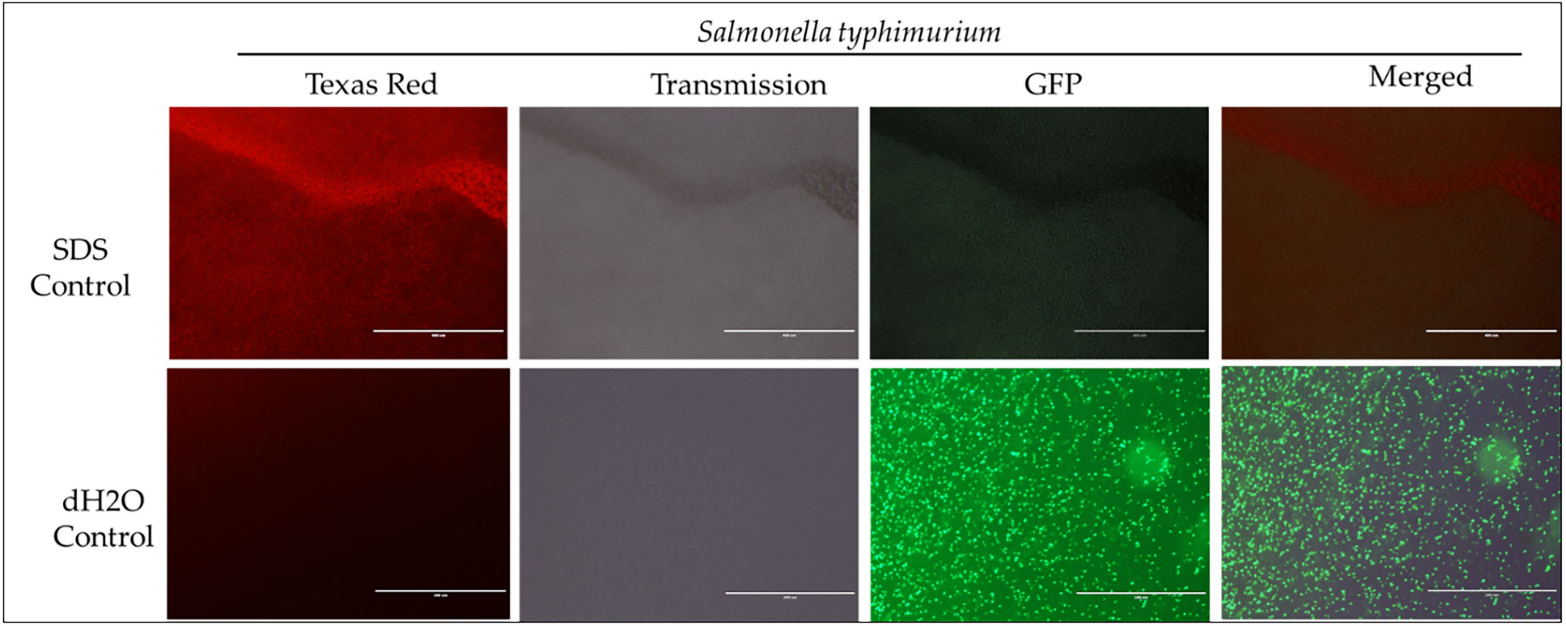
Immunofluorescence analysis of *S*. Typhimurium (ST) treated to 2% SDS as a positive control, while dH_2_O as a negative control. Scale bar = 100 μm. The immunofluorescence studies reveal that 2% SDS had killed *S*. Typhimurium (as shown in complete red fluorescence), whereas the dH_2_O revealed live bacteria growing in the sample as indicated by the green fluorescence.

**Figure 5:**
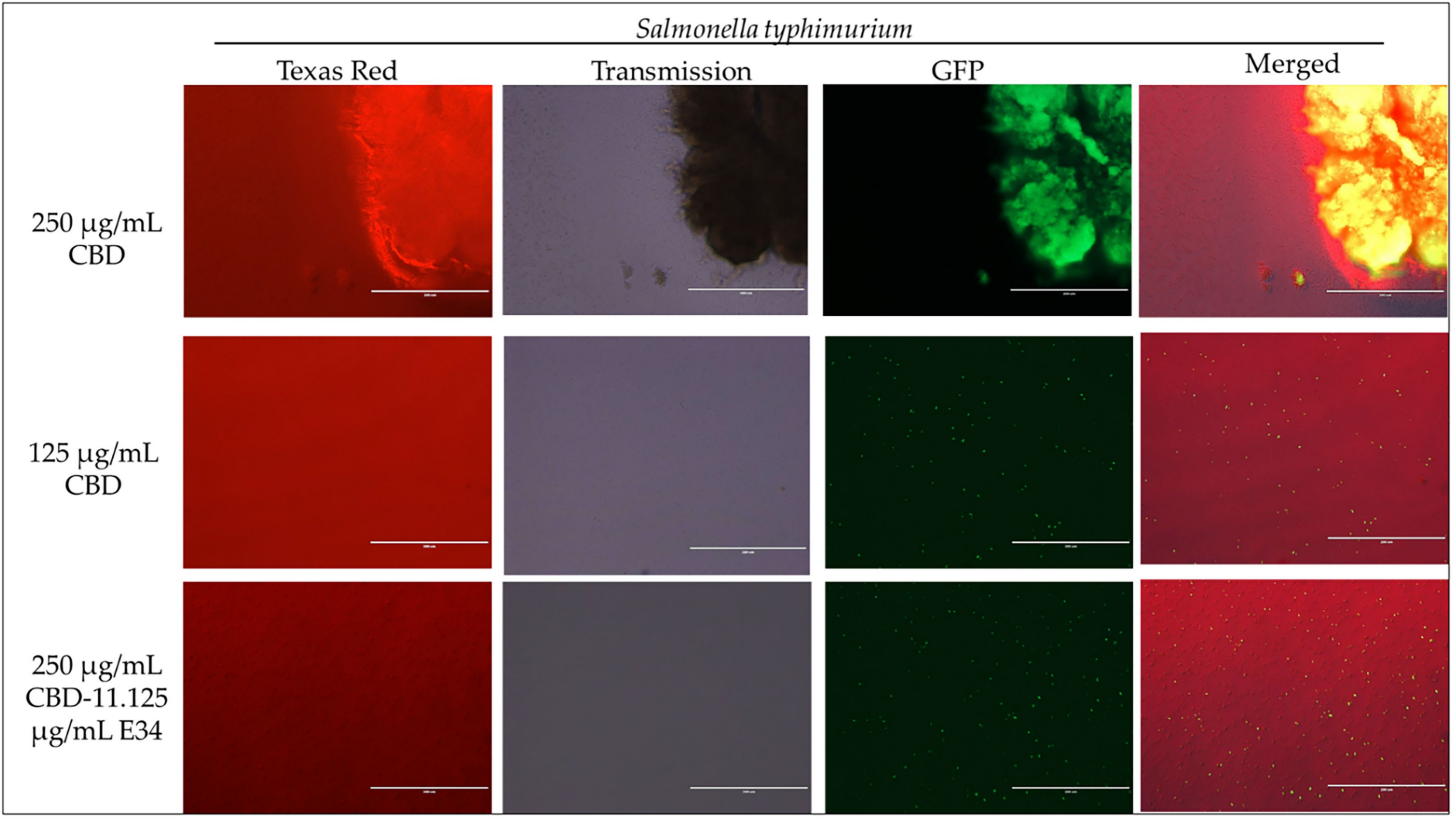
Immunofluorescence analysis of *S*. Typhimurium (ST) at high concentrations of CBD extract (250 μg/ml, 125 μg/ml, and 250 μg/ml + 11.125 μg/ml E34 TSP) treatment. As shown in all panels, treatment at higher concentrations killed the *S*. Typhimurium. However, bacteria seemed to cluster together into biofilms enhancing their ability to survive (See top panel).

**Figure 6:**
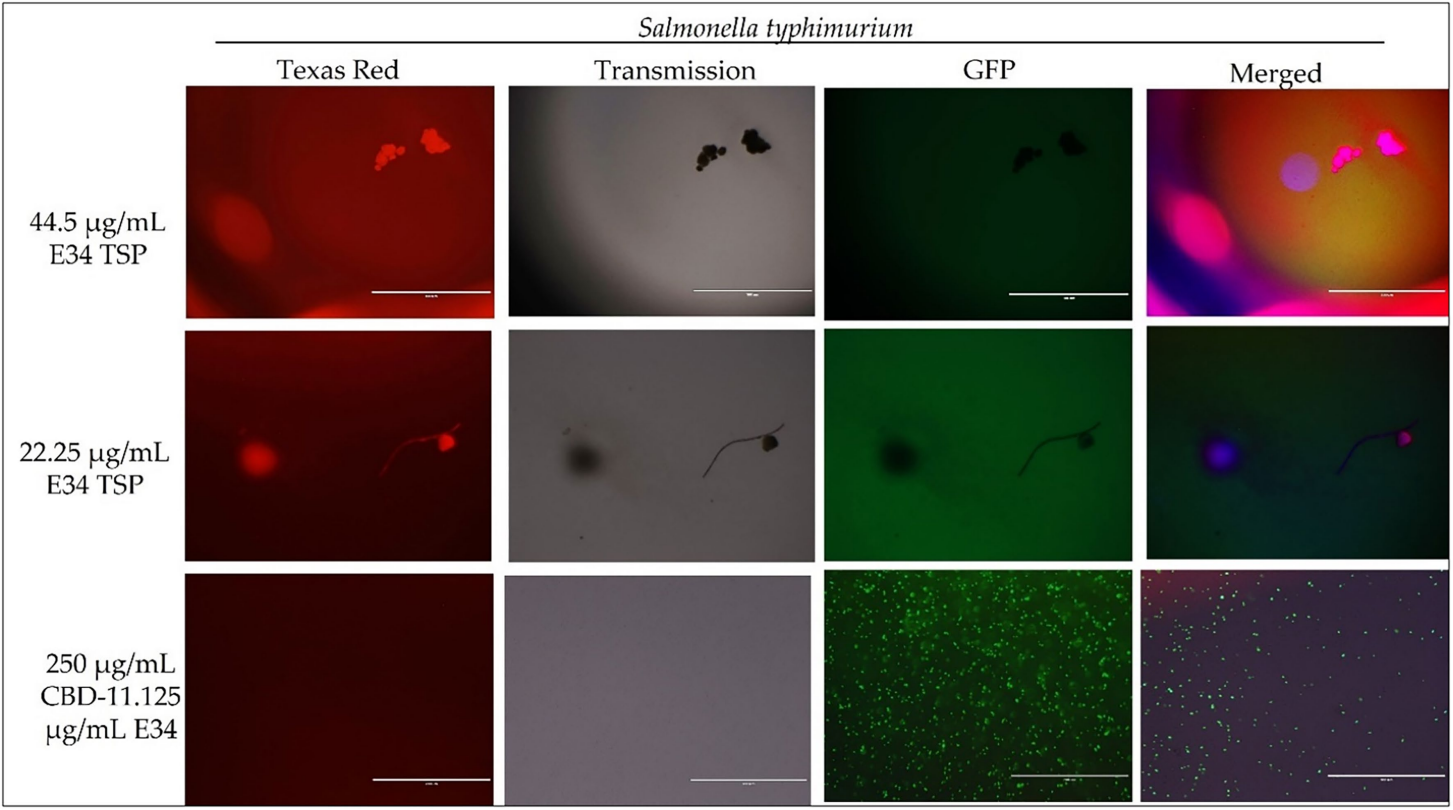
Immunofluorescence analysis of *S*. Typhimurium (ST) at higher concentrations of E34 TSP; 44.5 μg/ml E34 TSP, 22.25 μg/ml E34 TSP, and the combination treatment of 250 μg/ml CBD extract +11.125 μg/ml E34 TSP. As shown in all panels, treatment at higher concentrations killed the *S*. Typhimurium. However, bacteria seemed to survive the combination treatment of CBD extract and E34 TSP especially at the higher concentration of 250 μg/ml CBD extract.

**Figure 7:**
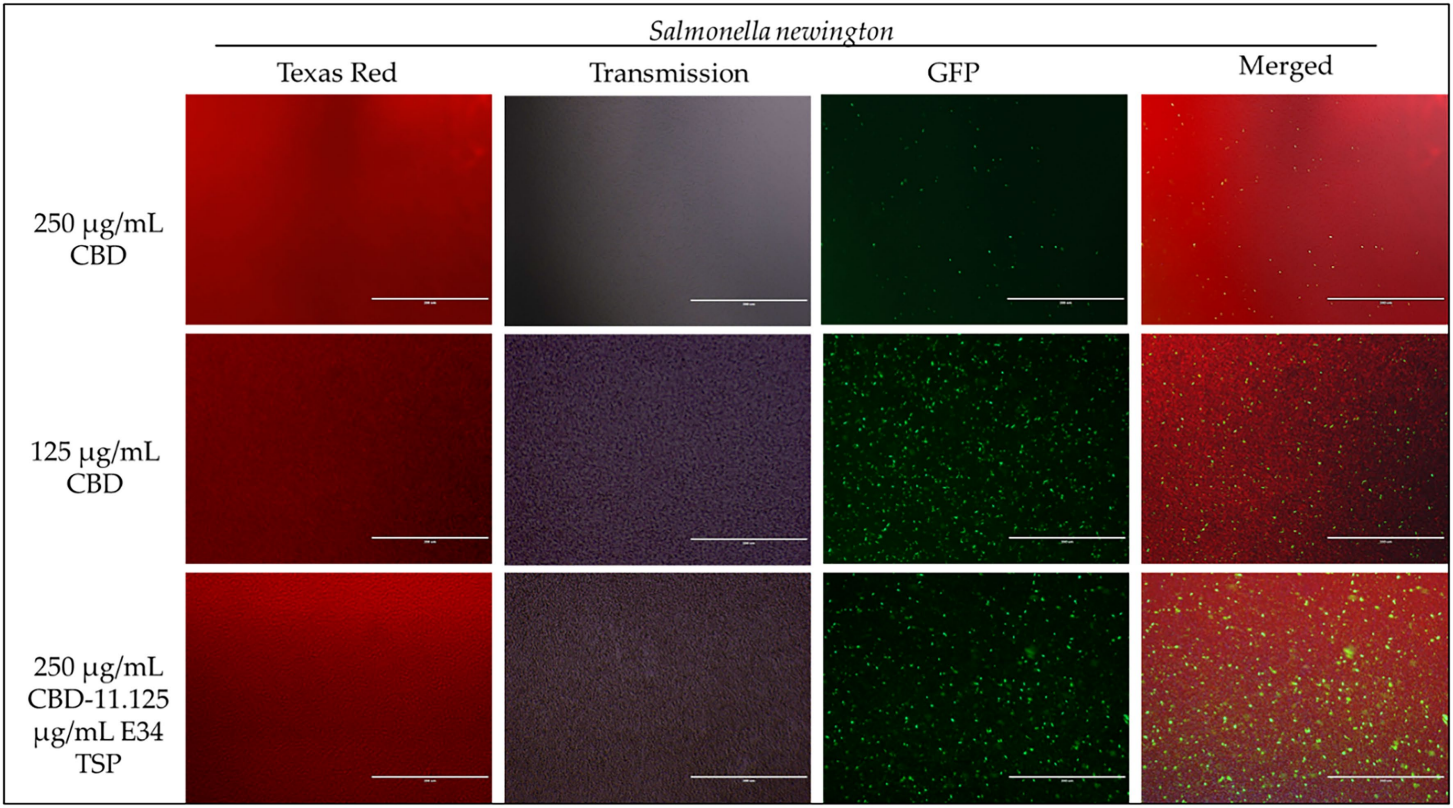
Immunofluorescence analysis of *S*. Newington (SN) at higher concentrations of CBD extract (250 μg/ml, 125 μg/ml, and 250 μg/ml + 11.125 μg/ml E34 TSP) treatments. Generally, there is observed higher killing of *S*. Newington in all treatments. Comparatively, there seems to be an observed lower killing of *S*. Newington at the combination treatment.

**Figure 8:**
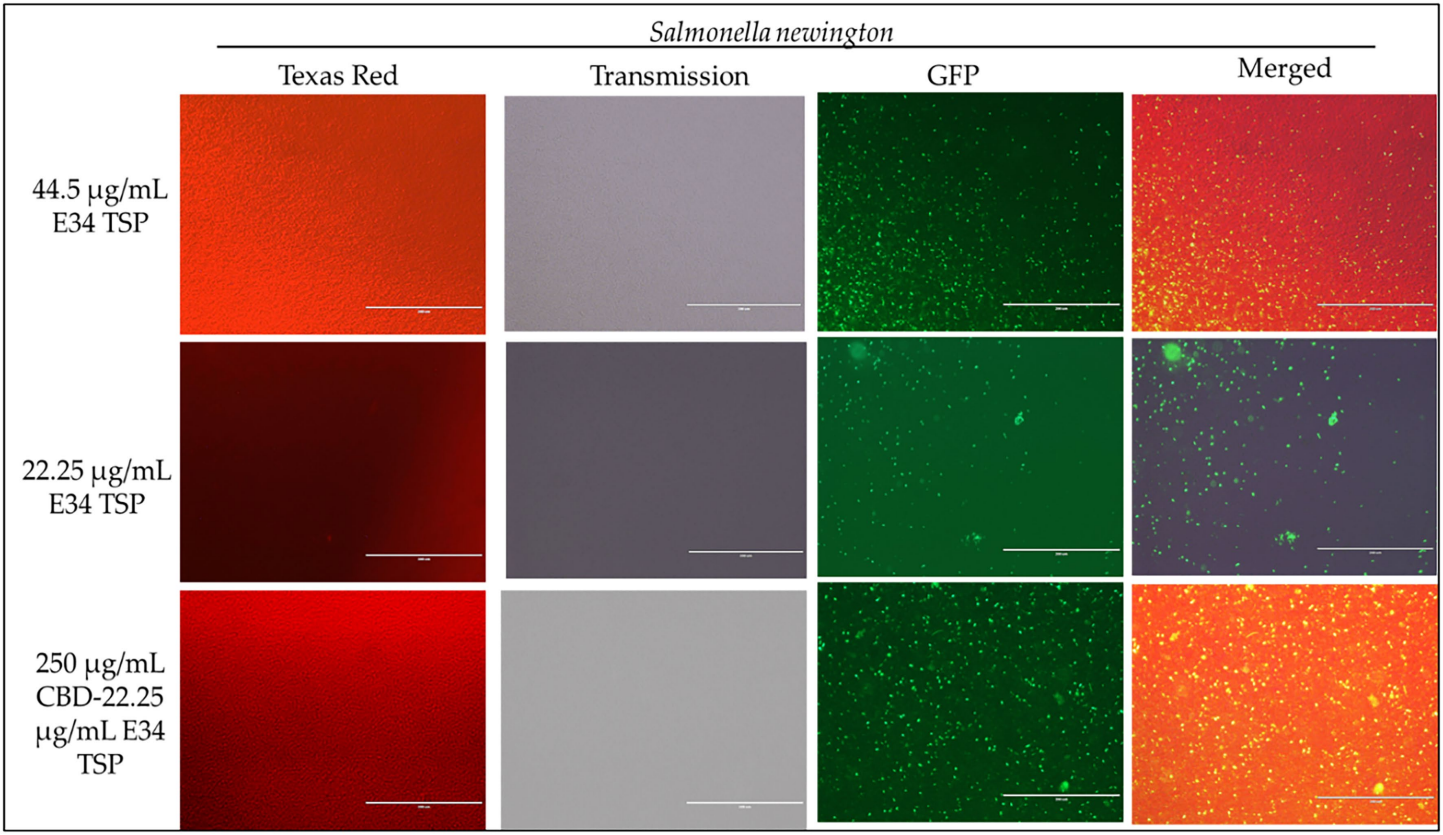
Immunofluorescence analysis of *S*. Newington (SN) treated to low concentrations of E34 TSP; 44.5 μg/ml, 22.25 μg/ml, and a combination treatment of 250 μg/ml CBD extract +22.25 μg/ml E34 TSP. The immunofluorescence images showed that the combination treatment (CBD extract and E34 TSP) had poorer membrane disruptive activities against *S*. Newington as compared to the monotreatment with E34 TSP.

To investigate the effect of E34 TSP, CBD extract, and CBD extract-E34 combination on *S*. Typhimurium and *S*. Newington viability through the disruption of the bacterial cytoplasmic membrane integrity, the membrane impermeable fluorescent DNA intercalating dye Propidium iodide (PI) and the membrane permeable green, fluorescent dye, SYTO-9 was employed. In brief, CBD resistant strains *S*. Typhimurium and *S*. Newington were grown to mid-log phase, diluted to cell concentration of 1 × 10^8^ and then treated with CBD extract, E34 TSP, or the combination of CBD extract and E34 TSP. Two controls, dH_2_O and 2% SDS served as the negative and positive controls, respectively. Figures 4–8 illustrates the effects of treatment on the viabilities of *S*. Typhimurium and *S*. Newington.

### 3.4. Time dependent analysis of E34 TSP and CBD extract treatments on *Salmonella* Typhimurium and *Salmonella* Newington growth at lag phase

To understand the effect of time kinetics of E34 TSP, CBD extract, or their combination treatment to *S*. Typhimurium and *S*. Newington, we subjected bacterial cells growing at lag phase (approximately 0.081 OD_600_) to E34 TSP and CBD extract treatment.

**Figure 9:**
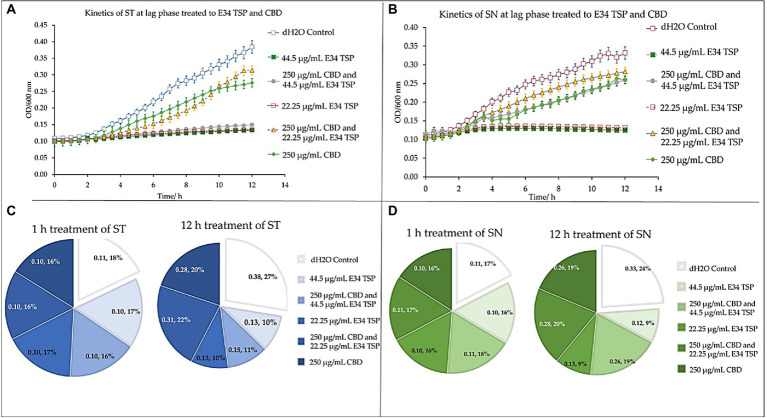
Kinetics of combined treatment of E34 TSP with CBD extract on **(A)** *S*. Typhimurium (ST) in the lag growth phase, **(B)** *S*. Newington (SN) in the lag growth phase. **(C)** Pie chart illustrating the relative percent changes in OD_600_ of *S*. Typhimurium among treatments. **(D)** Pie chart illustrating the relative percent changes in OD_600_ of *S*. Newington among treatments. Studies were initiated an initial OD_600_ of approximately 0.1 Data shown are from three independent experiments expressed as means ± SEM.

At the lag phase treatment, while all performed better than the control, treatment of *S*. Typhimurium to 44.5 μg/ml and 22.25 μg/ml of E34 TSP performed significantly higher in inhibiting the bacterial growth. The combination treatment of 250 μg/ml CBD extract and 44.5 μg/ml E34 TSP also performed significantly better in inhibiting the *S*. Typhimurium growth than the 250 μg/ml and 22.25 μg/ml combination treatment or the 250 μg/ml CBD extract monotreatment (Figure 9A). In Figure 9B however, only E34 TSP treatment at concentrations 22.25 μg/ml and 44.5 μg/ml showed high inhibition of *S*. Newington, all other treatments showed poor inhibition kinetics. Comparatively, as shown in Figure 9C, the control group as expected showed the highest increase in OD_600_ of 38% compared to the other treatments, followed by the 250 μg/ml CBD extract and 22.25 μg/ml E34 TSP combination treatment which recorded 22%. Thus, indicating that these two treatments performed the poorest in inhibiting *S*. Typhimurium growth at lag phase at the 12 h time point. The 22.25 μg/ml E34 TSP treatment showed the best inhibitory characteristic at the 12 h time point, recording 10%.

In treating *S*. Newington, as shown in Figure 9D, the best inhibitory effect again was observed in the 22.25 μg/ml E34 TSP treatment, which recorded a comparative rate of 16% at the 1 h time point and reduced this to 9% at the 12 h time point.

### 3.5. Dose dependent analysis of E34 TSP and CBD extract treatment on *Salmonella* Typhimurium and *Salmonella* Newington

**Figure 10:**
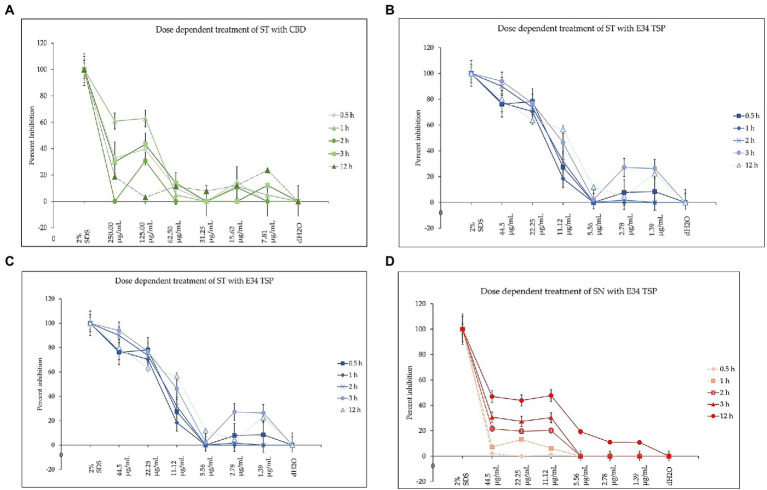
**(A)** The effect of CBD extract on *S*. Typhimurium presented as means ± standard deviation. Data shown are from three independent experiments expressed as means ± SEM. As can be seen, CBD extract although showed slightly higher inhibition at higher doses, it seemed not to exhibit time dependent inhibition of *S*. Newington. **(B)** The effect of E34 TSP on *S*. Typhimurium presented as means ± standard deviation. Data shown are from three independent experiments expressed as means ± SEM. As can be seen, E34 TSP seems to inhibit *S*. Typhimurium in both time and dose dependent nature. **(C)** The effect of CBD extract on *S*. Newington presented as means ± standard deviation. Data shown are from three independent experiments expressed as means ± SEM. As shown, CBD extract did not show any significant inhibition of *S*. Newington in all treatments. **(D)** The effect of E34 TSP on *S*. Newington presented as means ± standard deviation. Data shown are from three independent experiments expressed as means ± SEM. As can be observed, E34 TSP seems to inhibit *S*. Newington in both time and dose dependent nature.

To investigate how the concentration of E34 TSP, CBD extract or CBD extract-E34 TSP combinations could inhibit bacterial cell growth, we treated *S*. Typhimurium and *S*. Newington to varying concentrations of E34 TSP, CBD extract, and combinations of CBD extract -E34 TSP. Figures 10A–D show the inhibition curves at the various treatment doses.

### 3.6. E34 TSP treatment potentiate bacterial membrane disruption

**Figure 11:**
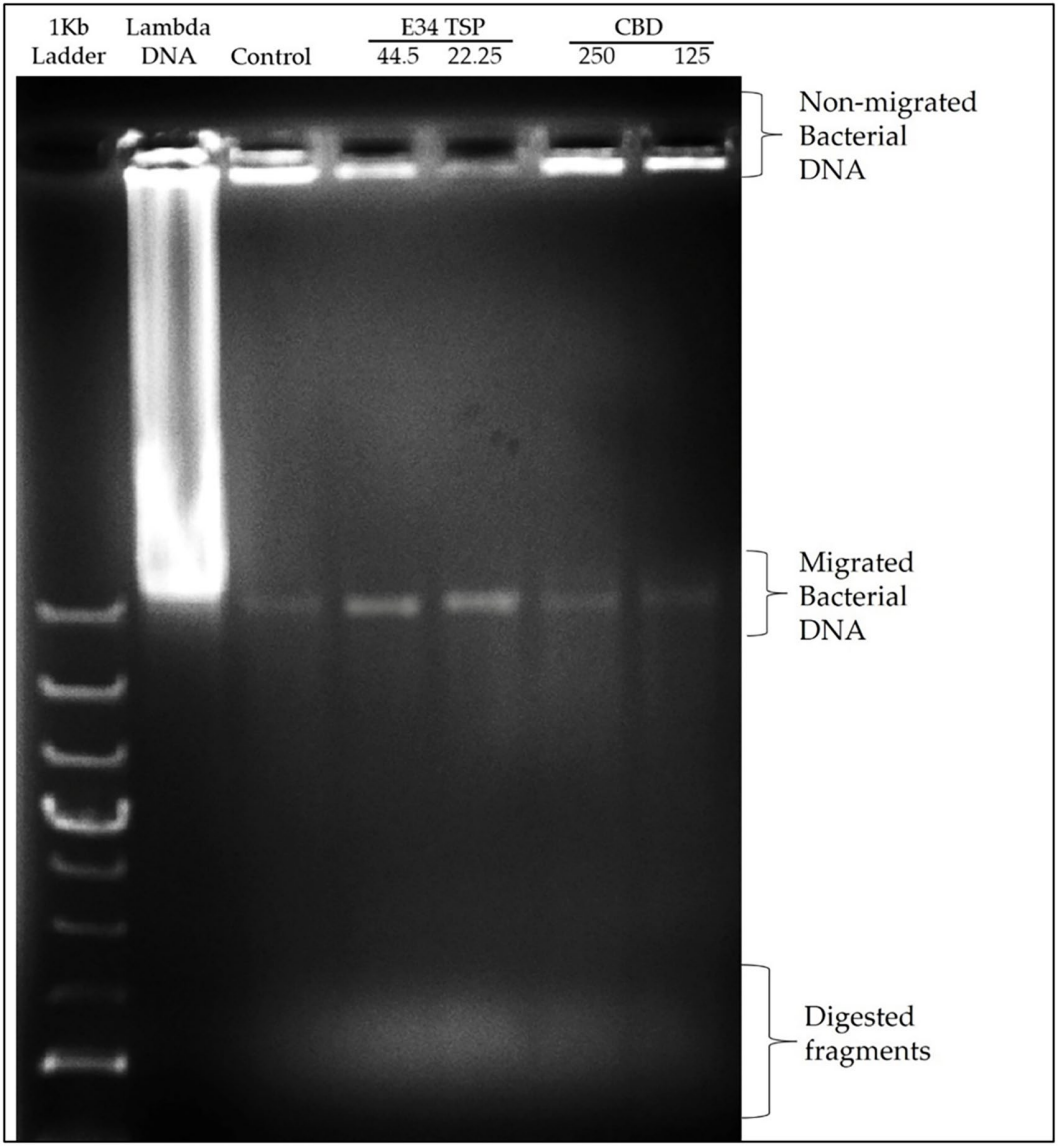
Agarose gel analysis of *S*. Typhimurium treated to CBD extract, or E34 TSP.

The nucleic acid migration analysis of *S*. Typhimurium which whole bacteria whole cells were treated to CBD extract, or E34 TSP as shown in Figure 11 indicates that E34 TSP causes lysis of *S*. Typhimurium cells.

### 3.7. Analysis of dehydrogenase activity of treated *Salmonella* Typhimurium and *Salmonella* Newington

The dehydrogenase of *Salmonella* is one critical reductase that is modulated by various metabolites, which include among others the glycolytic TCA cycle and related intermediates, such as PEP, pyruvate, acetyl CoA, citrate, etc. Another set of metabolites that actively regulate *Salmonella* dehydrogenase activity are the purine nucleotides (AMP, ADP, ATP, CAMP, GMP, GDP, and GTP). For instance, ATP is a potent activator of glutamate dehydrogenase (44). Thus, to assess the effect of CBD extract treatment to dehydrogenase of *Salmonella* spp. used in this study, we employed resazurin assay.

For *S*. Typhimurium, the effect of CBD extract on the bacteria dehydrogenase activity seems to negatively correlate with the concentration of CBD extract at the 1 h time point. E34 TSP showed no significant differences in all doses. The *S*. Newington strain showed lower relative values when treated to E34 TSP at all concentrations except at 2.78 μg/ml and 1.39 μg/ml at the 1 h time point. CBD extract at all concentrations showed no significant effect on *S*. Newington at 1 h time point. In general, 1 h post treatment of *S*. Typhimurium with CBD extract showed far lower dehydrogenase activity than E34 TSP treatments of the same strain. This is unexpected since our previous results demonstrated that E34 TSP showed higher killing of *S*. Typhimurium than CBD extract (Figure 3). This might be due in part to the inability of E34 TSP to inhibit the dehydrogenase enzyme in *S*. Typhimurium whereas CBD does inhibit it. A question remaining however, is why then we have less killing effect of CBD extract than E34 TSP in *S*. Typhimurium. To answer this, we infer that, there might be a possible alternative dehydrogenase pathway utilized by *S*. Typhimurium in its energy metabolism. In treating *S*. Newington however, E34 TSP treatments showed relatively lower dehydrogenase activity compared to the CBD extract treatment.

For *S*. Typhimurium, the effect of CBD extract and E34 TSP was not hugely different from each other except the 31.25 μg/ml CBD extract treatment which considerably limited the dehydrogenase activity. The treatment of *S*. Newington to E34 TSP however, showed general shutdown of the dehydrogenase enzyme at the 5 h time point. Interestingly, CBD extract treatment to the same strain produced slightly higher dehydrogenase activity as compared to the E34 TSP treatment. This trend is in consonance with the killing ability of E34 TSP on *S*. Newington than CBD does. All SDS controls showed the lowest activity indicative of dead bacteria. Comparing the 5 h treatment to 1 h treatment, all the samples showed significantly lower dehydrogenase activity at 5 h than 1 h time point. This is indicative of the killing ability of all the treatments.

## 4. Discussion

Due to the emergence of multidrug-resistant bacteria, the use of bacteriophage as an alternative to antibiotics has been reconsidered. However, this approach can be very effective when combined with other agents. The combination of these two agents can provide better bacterial suppression and lower the chances of the development of resistance. It also allows for more efficient penetration into the bacterial population. Although neutral effects have been observed in some studies, combined approaches are still considered important in preventing the development of multidrug-resistant bacteria (45). The main culprit for multi-drug resistance has been excessive use of antibiotics, which jeopardizes their effectiveness for controlling these pathogenic agents (46). Considering this, bacteriophages and their derivatives are becoming more widely accepted as viable complementary techniques for use in food safety, health, and medicine.

In our previous studies, we demonstrated the antimicrobial effect of CBD extract against the Gram-negative bacterium *S*. Typhimurium (8), and in a separate publication, we also revealed that CBD synergizes with ampicillin and polymyxin B in killing *S*. Typhimurium ((47)). E34 phage protected Vero cells from *Salmonella* infection (38). In this work, E34 phage’s LPS hydrolase, which is the E34 TSP, was expressed from a previously published clone (38), and used in combination with CBD extract to investigate the killing abilities of the two antimicrobial agents against two bacterial strains of *Salmonella*. The proceeding sections provides data obtained from multiple biological assays carried out to determine the antimicrobial activities of CBD extract and E34 TSP against CBD-resistant strains of *S*. Typhimurium and *S*. Newington.

E34 phage is a bacteriophage that infects *S*. Newington, and it uses its hydrolase machinery which is the tailspike protein (TSP) for the initial interaction and anchoring of the phage particle to the LPS of the cell and subsequent hydrolysis and anchoring of the LPS to the membrane of the bacteria. In this study, the E34 TSP was expressed under the control of the T7 promoter in pET30a-LIC vector, purified and combined with CBD as an antibacterial agent against CBD-resistant strains of *Salmonella*. Validation of the cloned insert was achieved *via* PCR reaction using two primers that amplified exactly the tailspike gene, gp19 in the clone. An agarose gel electrophoresis of PCR product demonstrated that it carried the exact size of 1.818 kbp insert (Figure 1A). Non-recombinants control did not yield any band, indicative of the absence of the insert. Cobalt-NTA column was used for affinity purification *via* FPLC (chromatograph shown in Figure 2), and samples were run on SDS PAGE to validate the purity of the protein.

The ability of CBD extract to potently inhibit *S*. Typhimurium was previously demonstrated in our laboratory (8), other works by (48) also revealed that CBD is a novel antibiotic adjuvant that potentiates the effect of the bacitracin against Gram-positive bacteria (e.g., *Staphylococcus aureus*, *Listeria monocytogenes*, and *Enterococcus faecalis*). The same group in another study explored the mechanism of resistance to CBD by *Staphylococcus aureus*. They discovered through resistant strains genome sequencing that the farE/farR system encoding a fatty acid efflux pump (FarE) and its regulator (FarR) were mutated, which showed diminished susceptibility to both CBD and bacitracin (35).

Proceeding from lag phase, bacteria enter log phase, a phase characterized by exponential growth. One major biological characteristic of this phase is the high metabolic activities occurring, which is due in part to high DNA replication, RNA translation, cell wall biosynthesis, and in part due to high cell division. While bacteria are metabolically hyperactive at this phase, they are also most vulnerable too, it is in this phase that antibiotics and other antibacterial agents can produce their highest potency. Usually, most of these agents target bacteria cell wall synthesis (e.g., Beta-lactams), or protein synthesis (e.g., thermorubin, (49)), DNA transcription (e.g., Anthracyclines (50)), and RNA translation (e.g., spectinomycin (51)). In this work, we investigated the effect of treating *S*. Newington and *S*. Typhimurium to CBD extract, E34 TSP, and the combination of the two agents at both early and late log phase of the bacteria. As shown in Figure 3 while generally all treatments performed better in *S*. Newington than in *S*. Typhimurium, the E34 TSP treatments gave the best inhibition than both CBD extract and CBD extract-E34 TSP combination. While it is predictable that E34 TSP monotreatment will perform better than CBD extract in reducing the bacterial growth, since these strains were CBD resistant, it is surprising that the combination treatment performed poorly. While these results necessitate a multiple dose analysis of CBD extract, and E34 TSP combinations, it seems safe to infer that there is probably an unknown interaction between the CBD extract and E34 TSP. This might be due in part to CBD in the CBD extract probably binding to the catalytic site of the E34 TSP, thus blocking it from its endorhamnosidase activity, however this observation does not answer for lower concentration of E34 TSP in combination with CBD extract which showed higher bacterial OD_600_. Since these strains are CBD-resistant, inactivated E34 TSP and high amount CBD extract will possibly still show minimal effect on the bacteria, and such should be highly pronounced at the lower E34 TSP concentrations. Figures 4–8 depict the immunofluorescent images showing the ability of E34 TSP to kill *S*. Typhimurium and *S*. Newington. However, as shown in Figure 5, the bacteria seemed to pool into micro clusters in high CBD concentrations that possibly provided unique shields against the CBD. This clustering might enable the development of biofilm by the bacteria, thus enhancing their CBD resistance (52). Micro clustering enables quicker cell–cell communication in the form of quorum sensing or membrane vesicle trafficking. The membrane vesicles in bacteria play a role in cell–cell communication between bacteria themselves and between bacteria their hosts. Membrane vesicles are an important component of anti-bacterial resistance and thus have gained relevance in antibacterial resistance research. Given this, (53) revealed that CBD affected the membrane vesicle profile and membrane vesicle release of bacteria. They reported that CBD had a strong inhibitory influence on membrane vesicle release of Gram-negative bacteria such as *E. coli* VCS257, and negligible inhibitory influence of CBD on Gram-positive bacteria such as *S. aureus* subsp. *aureus* Rosenbach 1884 membrane vesicle release. Thus, the micro clustering might be a defensive maneuver to overcome CBD anti-vesicle release property.

The commencement of disease symptoms mostly starts after the lag phase of bacterial infection. This initial phase of the bacterial growth is characterized by cellular activity that typically involves the synthesis of proteins without much growth. To understand the effect of CBD on our CBD-resistant strains growing at lag phase, we treated *S*. Typhimurium and *S*. Newington to CBD, E34 TSP and CBD-E34 TSP combination. As it is well known, CBD is recognized as a potent antimicrobial. (54) Studied the antimicrobial characteristics of CBD-resistance in *Staphylococcus aureus*, *Streptococcus pneumonia*, and *Clostridioides difficile*. Their findings suggested that CBD has an impeccable impact on biofilm and topical *in vivo* efficacy. In this study, at the lag phase, while all treatments performed better than the control, monotreatment of *S*. Typhimurium and *S*. Newington to 44.5 μg/ml and 22.25 μg/ml of E34 TSP performed significantly higher in inhibiting the bacterial growth. The results corroborated with the data obtained in the log phase treatment indicating that bacteria growth phase did not significantly affect the performances of the treatments.

As shown in Figure 10A, CBD showed slightly higher inhibition at higher doses, but did not exhibit time dependent inhibition of *S*. Newington. E34 TSP demonstrated both time and dose dependent inhibition of *S*. Typhimurium (Figure 10B). As expected, CBD extract failed to show any significant inhibition of *S*. Newington in all treatments (Figure 10C). Treatment of *S*. Newington to E34 TSP showed time and dose dependent inhibition (Figure 10D).

Given that there was observed substantial inhibition of *S*. Typhimurium and *S*. Newington caused by E34 TSP on the CBD-resistant strains, we found it important to investigate the possible mechanism of the bacterial killing. We reasoned that it might be due to membrane integrity disruption. For this reason, we analyzed E34 TSP and CBD extract treated samples of CBD-resistant *S*. Typhimurium using 0.8% agarose gel. Previous works by (48) revealed that CBD is an effective helper compound in combination with bacitracin to kill Gram-positive bacteria *via* possible membrane disruption. Furthermore, (8) also demonstrated the CBD extract in combination with polymyxin B was potent bactericidal agent whose mechanism of action was membrane lysis. In this study, treatment of CBD resistant strains of *S*. Typhimurium to CBD extract produced relatively denser bands than the control group as indicated in Figure 11. The densities of the bands designated as non-migrated bacterial DNA showed highest in the Control group, followed by the CBD extract treatment group, E34 TSP treatment group showed the lowest density of bands in the non-migrated bacterial DNA. This points to the possibility that E34 TSP caused the highest bacterial membrane lysis, thus genomic content was free to migrate out of the cells. As depicted in the Migrated Bacterial DNA, the E34 TSP treated samples also gave the highest densities compared to both the control group and the CBD extract treated samples. Another interesting observation was the presence of short DNA fragments. These fragments were due to possible endonuclease activity during the treatment administration. As can be observed in Figure 11, most of these fragments are centered on the E34 TSP treated lanes, indicating further the possibility of E34 TSP exerting a much higher lytic activity on *S*. Typhimurium than CBD. The inability of CBD extract to exert high lytic action on *S*. Typhimurium could be due in part to the resistance of these strains to CBD. We infer that, the resistance mechanism is possibly *via* membrane content modifications that enforced lesser susceptibility to CBD interactions. Furthermore, CBD has been demonstrated to show potent membrane disruption as its primary mechanism of attack (54). Intuitively, the same study also hinted for the first time how CBD deactivates the “urgent threat” pathogen *Neisseria gonorrhoeae*. Similarly, (55), reported how CBD extract was effective against *Neisseria gonorrhoeae*, *Neisseria meningitides*, *Moraxella catarrhalis*, and *Mycobacterium tuberculosis*. Their CBD formulation, however, was in combination with polymyxin B which is a known antibiotic that shows very potent membrane disruption activity (56). They stated that the activities were achieved at a polymyxin B concentration of ≤2 μg/ml and CBD concentration of ≤ 4 μg/ml, especially against *Klebsiella pneumonia*, *Escherichia coli*, and *Acinetobacter baumannii*. In our research work, we showed that CBD caused very slight membrane disruption compared to E34 TSP which caused a much higher membrane disruption (Figure 11). The lower membrane disruption of CBD extract might be attributable to the resistant strains used for the study.

**Figure 12:**
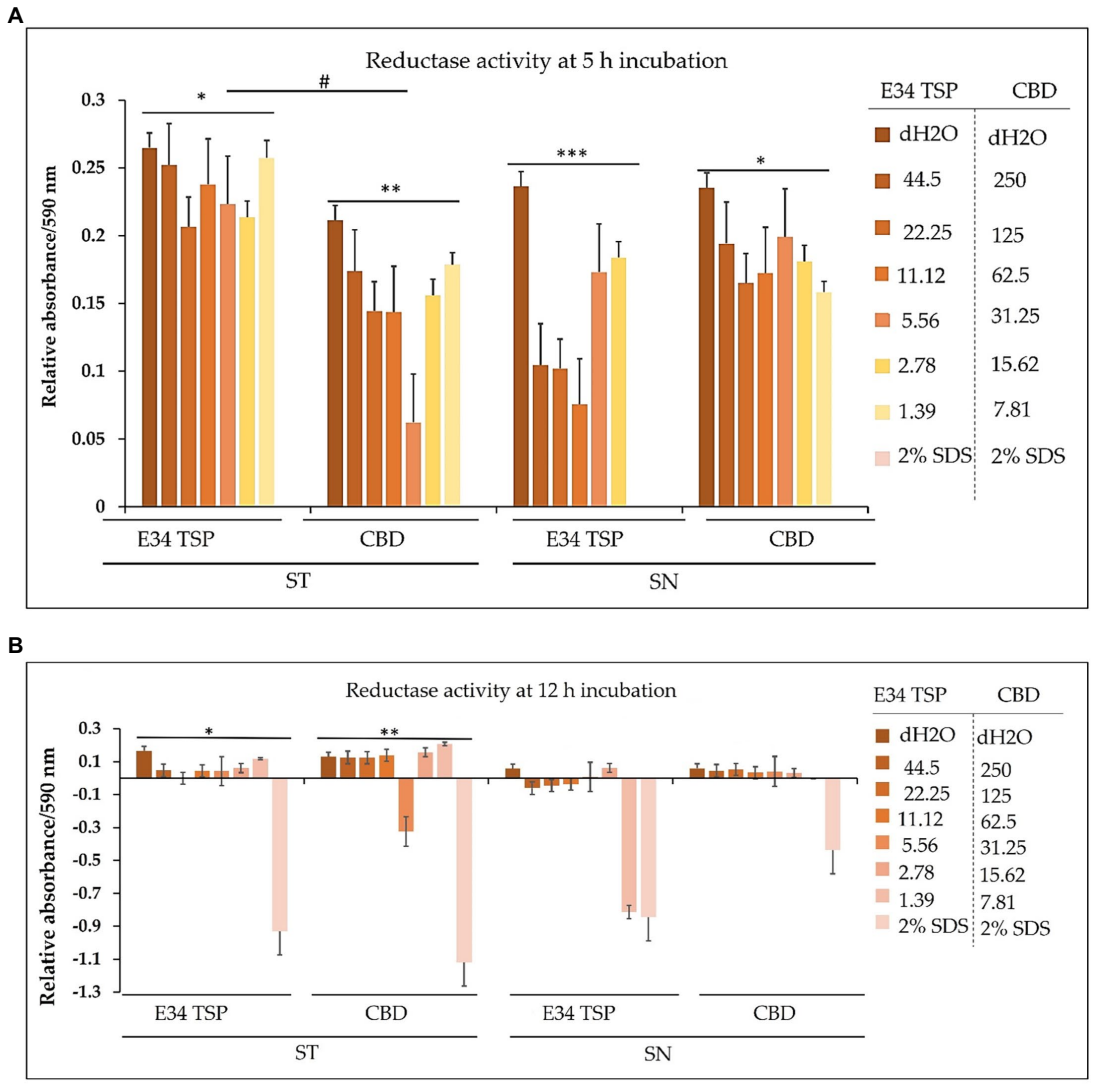
**(A)** Chart illustrating the *S*. Typhimurium and *S*. Newington dehydrogenase activity in 1 h after treatment to varying concentrations of CBD extract or E34 TSP. Data shown are from three independent experiments expressed as means ± SEM. * and ** value of *p*s ≤ 0.0000024, *** and ****value of *p*s ≤ 0.0032, # *p*-value = 0.0038. **(B)** Chart illustrating the *S*. Typhimurium and *S*. Newington dehydrogenase activities in 5 h after treatment to varying concentrations of CBD extract, or E34 TSP. Data shown are from three independent experiments expressed as means ± SEM. * and ** *p*-values ≤ 0.000367.

In bacteria, respiratory chains are composed of various types of transport constituents, such as cytochromes, quinones, iron–sulfur proteins, and flavoproteins. The differential transport of protons and electrons through the cytoplasm leads to the formation of a proton gradient membrane. This membrane can be used to drive the formation of ATP through the F1/F0 ATPase (57–59). Electrons are transported through a variety of redox carriers to reach the oxygen-rich environment during respiration. They can also be obtained from alternative terminal electron acceptors when oxygen is unavailable (60). The glutamate dehydrogenase of *Salmonella* is one critical reductase that is modulated by various metabolites, which include among others the glycolytic, TCA cycle and related intermediates, such as PEP, pyruvate, acetyl CoA, citrate, etc. Another set of metabolites that actively regulate *Salmonella* glutamate dehydrogenase activity are the purine nucleotides (AMP, ADP, ATP, CAMP, GMP, GDP, and GTP). For instance, ATP is a potent activator of glutamate dehydrogenase (44). Thus, to assess the effect of CBD treatment to dehydrogenase of *Salmonella* spp. used in this study, we employed resazurin assay. As shown in Figure 12A, the effect of CBD on *S*. Typhimurium dehydrogenase activity seems to negatively correlate with the concentration of CBD at the 1 h time point whereas E34 TSP showed no significant difference in all doses. In general, 1 h posttreatment of *S*. Typhimurium with CBD showed far lower dehydrogenase activity than E34 TSP treatments of the same strain. This is unexpected since our previous results demonstrated that E34 TSP showed higher killing of *S*. Typhimurium than CBD (Figure 3). This might be explained in part by proposing that E34 TSP does not interact with dehydrogenase enzyme in *S*. Typhimurium whereas CBD does possibly interact with the enzyme. The concern, however, is why the less killing effect of CBD observed in *S*. Typhimurium than E34 TSP. To answer this, we infer that there might be a possible alternative dehydrogenase pathway utilized by *S*. Typhimurium in energy metabolism especially at the 1 h time point. In treating *S*. Newington however, E34 TSP treatments showed relatively lower dehydrogenase activity compared to the CBD treatment.

As shown in Figure 12B, treatment of *S*. Typhimurium and *S*. Newington showed a general decrease in dehydrogenase activity in 5 h. The most pronounced decrease however was observed in *S*. Newington treated to E34 TSP which demonstrated a complete inactivation of the enzyme at the 5 h time point. Interestingly, CBD treatment to the same strain produced slightly higher dehydrogenase activity as compared to the E34 TSP treatment. This trend is in consonance with the killing ability of E34 TSP on *S*. Newington than CBD does. All SDS controls showed the lowest activity indicative of dead bacteria. Comparing the 5 h treatment to 1 h treatment, all the samples showed significantly lower dehydrogenase activity at 5 h than 1 h time point. This is indicative of the killing ability of all the treatments.

To halt nosocomial infections, intensive research on antimicrobial surfaces is necessary. Currently, bacteriophages are emerging as alternative candidates for antimicrobial surfaces (61). Bacteriophages or their products can be selective in the destruction of bacteria since they are their natural enemies. More interesting is their host specificity which allows the bacteriophages avoid killing the human host’s microbiota if used as therapy (22). In this study, E34 TSP has been demonstrated to be effective in killing two CBD-resistant strains of *Salmonella* (*S*. Typhimurium and *S*. Newington). Although much in-depth studies are required to ascertain both the pharmacodynamics and pharmacokinetics of E34 TSP, most phages or their products are self-limiting, and show quick clearance from the body (62), thus, if E34 TSP is formulated into a drug, it could be beneficial in treating most *Salmonella* infections. The added benefits of using the E34 phage tailspike protein instead of the whole bacteriophage is because most whole phages can elicit strong immune response and have narrow host range, therefore making it difficult to be used as antibacterial agents. In this work, these challenges are eliminated, since only the spike protein is used. The mechanisms of action of the E34 TSP include the binding of tailspike protein to bacterial surface proteins, e.g., the outermembrane protein A (ompA) as well as the LPS of the bacteria (38). This protein is responsible for processing through and hydrolyzing the LPS of the bacteria (63). We have demonstrated that the E34 TSP showed broader spectrum of activity since it showed antibacterial activity against *S*. Typhimurium which is not E34 phage’s host. Future studies will include investigating the effectiveness of this protein in killing other bacterial strains unrelated to *Salmonella* to ascertain the spectrum of activity of the protein.

## 5. Conclusion

Given the rapid antimicrobial resistance development, it is crucial to look for other avenues to treat antimicrobial resistant bacteria. In our previous communication, we demonstrated the effectiveness of CBD as an antibacterial agent against *Salmonella* even at very low concentrations. We however observed that *Salmonella* developed resistance. In this work, we demonstrated that even strains that are resistant to the potent CBD could still be inhibited by a phage protein such as E34 TSP. Another important observation was the ability of the tailspike protein of E34 phage that is specific for only *Salmonella* Newington, showing antibacterial efficacy against *Salmonella* Typhimurium which is not its host. This is an interesting finding especially for health application since, this protein has indicated it ability to be used against multiple *Salmonella* strains, possibly indicating its broad-spectrum property. We also showed that the inhibition of the bacteria by E34 TSP was due in part to membrane disruption, and dehydrogenase inactivation by the protein. A CBD-E34 TSP combination treatment resulted in lower killing ability of the treatment. This finding indicates that CBD might have had an unknown interaction with the protein which may cancel their individual or combine efficacy in killing the bacteria. Nonetheless, further research is needed to fully elucidate the mechanism of action of E34 TSP only, E34 TSP and CBD extract combination in the killing of *Salmonella*. Furthermore, it will be very interesting to explore the genetic basis of the resistance development of these two strains of bacteria to CBD. In conclusion, this work highlights the crucial role phage protein such as E34 TSP could play in pathogenic bacterial control.

## Data availability statement

The original contributions presented in the study are included in the article/supplementary material, further inquiries can be directed to the corresponding authors.

## Author contributions

JA: conceptualization. II and JA: methodology, software, visualization, and writing–original draft preparation. JA, OA, and MS-F: validation and resources. JA, JX, MS-F, and OA: formal analysis. II, JA, AA, JX, MS-F, and OA: investigation. JA, JX, and OA: data curation. II, JA, JX, and OA: writing–review and editing. JA and OA: supervision. OA: project administration and funding acquisition. All authors contributed to the article and approved the submitted version.

## Funding

This research was funded by United States Department of Education, Title III-HBGI-RES.

## Conflict of interest

The authors declare that the research was conducted in the absence of any commercial or financial relationships that could be construed as a potential conflict of interest.

## Publisher’s note

All claims expressed in this article are solely those of the authors and do not necessarily represent those of their affiliated organizations, or those of the publisher, the editors and the reviewers. Any product that may be evaluated in this article, or claim that may be made by its manufacturer, is not guaranteed or endorsed by the publisher.
